# Dental Fogyism

**Published:** 1864-07

**Authors:** 


### DENTAL FOGYISM.

Read before the Brooklyn Society of Dental Surgeons.

BY DR. MARVIN.

You may think this a strange subject, gentlemen, to be made the basis of a paper and presented to this Association. So did I when the thought was first introduced into my mind, but the more I reflected on it, the more practical became the shape it assumed, and I discovered in the field which it opened before me much that, in my judgment, it would be interesting and perhaps profitable to consider.

We often hear the terms "old fogy" and "old fogyism" applied to people and systems of ideas, but we seldom hear any one *define* the terms, and the fact is that we are very frequently left to decide for ourselves what *they* actually mean who have used them.

Let me define what I understand is meant by the term. By "old fogyism" we mean that tenacious adherence to old ideas, old methods, old paths, which can not see any virtue in new ones, and refuses to examine them. It adheres to every thing old *because* it is old, and denounces everything new *because* it is new. Arguments effect nothing against it; it flings them off as readily as the mailed side of an iron-clad repels the whizzing ball. In the estimation of old fogy-ism, novelty is fatal to any system, which owns it as its characteristic. Present it as favorably as you may, the verdict is inevitable. It is satisfied with the old-fashioned way, and wants nothing to do with any new fashion.

Now, if there be "*old* fogyism" in the world, there must of necessity be *new* fogyism, else we could not, with propriety, use the word. We say "old fogy" to distinguish him whom we thus designate, from some opposite character. If old fogyism signifies an unyielding adherence to *old* views, new fogyism must signify the same regard for new ideas.

Fogyism, then, bereft of all prefixes, is simply an obstinate prejudice in favor of chosen views. This deduction is inevitable, and we must accept it as truth. Fogyism is every where prevalent, but it is not my province to search for it any where but within the limits of our own profession. Fogyism in dentistry, or, as I have named it at the beginning of this paper, Dental Fogyism, claims our attention.

There is no profession without it, hence we need not be surprised that we find it in dentistry. Finding it, therefore, we have only to consider its character and effects, and if good, commend, if evil, condemn them.

There are few, if any sciences, in which there have been more improvements during the past fifty years than in the science of dentistry. What, at that period, seemed a great achievement would to-day excite a smile of ridicule. Operations, which, at that time, were not deemed practicable, are now performed daily, with ease and uniform success; while others, not then conceived of enter into the list of accomplishments of many a practitioner. *Methods* of operation, also, are now practiced and considered indispensable, which, at that period, were unknown. New instruments and inventions of various kinds, calculated to promote facility of operation, have been introduced. In short, the instrument table and laboratory of a dentist of 1800 would form a great contrast if placed beside those of a dentist of 1863.

Besides this, the advance in dental knowledge has been very great. Not as great as the advance in dental skill, for it is too true of our profession that we are not a *studious* class. We are too apt to discard theoretical knowledge as useless, and consider the *practical* as only valuable. Still, despite this unfortunate trait, the advance in knowledge—in theoretical knowledge—in the knowledge which comprehends theory and understands how to reduce it to practice—has been very great. And to-day we can number among those who follow the profession of dentistry men of large attainments—men of well-stored minds—men who can give an intelligent reason for what they do.

Now there is no doubt that the old ways possessed some virtue, and that there was merit in the ideas, some of them at least, which prevailed in days gone by; but what would be the result of a comparison with the ideas and the knowledge of our enlightened day? We may ask this question without arrogating to ourselves greater mental ability than our fathers. The question does not pre-suppose this. The spirit of the age is progress. Men's minds are directed to the promotion of the growth and advancement of that which they make the subject of thought. And with all the results before us, which have been reached by thoughtful, studious minds, with the books, the improvements, the almost numberless aids which this age supplies, we should be justly chargeable with culpable neglect; nay, with mental inferiority, if we failed to show greater proficiency than they who lived and acted fifty years ago.

They made the most of their advantages; they lived up to the privileges they possessed, and so must we to be equal to them. And if we do this, if we manifest the same diligence which characterized them, and no more, our results must be far greater, far more distinguished than theirs could have been.

Now "old fogyism" would frown upon the improvements now in use, and condemn them; and if its voice prevailed we should be left in the possession of the same old clumsy instruments, and follow the same old awkward methods, which were peculiar to a half centnry ago. This would cripple all efforts at growth, would effectually check all advancement. Fogyism is the same to-day it ever was. Whether old or new it is an unworthy sentiment.

He is unwise who, in the pride of his own attainments, and the vain confidence of his own judgment, ventures to pronounce sentence of unworthiness upon the thoughts of others. He may prefer his own way; he has a right to do so; but he has no right to denounce another's. By so doing he may injure himself, and no man has more right, really, to injure himself than to injure another. He may throw away opportunities of acquiring knowledge which is valuable and which would greatly benefit him. And he may injure the profession, and no man has a right to do this. If he be a man of great mental endowments, he may catch an idea from some humble member of the profession, which will form the groundwork of the most valuable discovery. It may come in a crude form; if so, he, by his greater ability, may mould it and fashion it, and make it to become of the utmost importance to his art.

We are not perfect beings. No man has attained the utmost limit of knowledge in any branch. Let him not think so. Improvement is always possible. No one man can do all the thinking in the world; no one mind can exhaust any scientific subject; no one intellect can grasp the entire range of scientific development, nor sound to their lowest depths the hidden mysteries of any occult theme. If he would attain great elevation, let him ever be a student. The wisest men are always learners. And it is no sure evidence of large wisdom to disparage the ideas of others. Fogyism does this, and therefore let us abjure fogyism now and forever. What we have attained that is valuable, let us adhere to; but let us reach out for higher attainments. Even blocks of wood rubbed together vigorously, produce fire; so the minds of men, brought into contact, give forth new energies, and produce new results.

Fogyism is the enemy of association. Association is the progenitor of improvement. Wisdom says, cultivate association, intelligent association. Let us hear her voice and be obedient to her counsels. Then we may expect to reap benefit; otherwise, never.

It is unnecessary to recite instances of fogyism. We have all met them; each one knows when and where. I treat of the general subject, and I think it must be admitted by every candid mind to be a belittling idea, an unworthy sentiment. It should have no place in so noble a branch of the restorative art as dentistry. It can have no place in noble minds. As the foe to our progress we must give it battle, and as we wish to become eminent and increasedly useful, we will cherish no fellowship with a sentiment that cramps our energies and dwarfs our attainments. No dentist can hope for an upward and shining pathway, until he has divorced himself at once and forever from fogyism.

